# Research on UAV Robust Adaptive Positioning Algorithm Based on IMU/GNSS/VO in Complex Scenes

**DOI:** 10.3390/s22082832

**Published:** 2022-04-07

**Authors:** Jun Dai, Xiangyang Hao, Songlin Liu, Zongbin Ren

**Affiliations:** 1 Institute of Geospatial Information, Information Engineering University, Zhengzhou 450001, China; daijun502@163.com (J.D.); lsl759514@126.com (S.L.); rzb13017600350@163.com (Z.R.); 2 School of Aerospace Engineering, Zhengzhou University of Aeronautics, Zhengzhou 450001, China

**Keywords:** UAV, robust adaptation filter, multi-source fusion, error state Kalman filter (ESKF), information sharing coefficient

## Abstract

As an important component of autonomous intelligent systems, the research on autonomous positioning algorithms used by UAVs is of great significance. In order to resolve the problem whereby the GNSS signal is interrupted, and the visual sensor lacks sufficient feature points in complex scenes, which leads to difficulties in autonomous positioning, this paper proposes a new robust adaptive positioning algorithm that ensures the robustness and accuracy of autonomous navigation and positioning in UAVs. On the basis of the combined navigation model of vision/inertial navigation and satellite/inertial navigation, based on ESKF, a multi-source fusion model based on a federated Kalman filter is here established. Furthermore, a robust adaptive localization algorithm is proposed, which uses robust equivalent weights to estimate the sub-filters, and then uses the sub-filter state covariance to adaptively assign information sharing coefficients. After simulation experiments and dataset verification, the results show that the robust adaptive algorithm can effectively limit the impact of gross errors in observations and mathematical model deviations and can automatically update the information sharing coefficient online according to the sub-filter equivalent state covariance. Compared with the classical federated Kalman algorithm and the adaptive federated Kalman algorithm, our algorithm can meet the real-time requirements of navigation, and the accuracy of position, velocity, and attitude measurement is improved by 2–3 times. The robust adaptive localization algorithm proposed in this paper can effectively improve the reliability and accuracy of autonomous navigation systems in complex scenes. Moreover, the algorithm is general—it is not intended for a specific scene or a specific sensor combination– and is applicable to individual scenes with varied sensor combinations.

## 1. Introduction

With the rapid development of research on autonomous and intelligent unmanned systems, UAVs can now operate in high-risk and complex environments, thus expanding the scope for human activities by virtue of their flexibility, low cost, and strong adaptability. Therefore, research on their application is of great significance to the military and civilian fields [1,2,3].

At present, sensors that can be used for autonomous navigation and positioning include inertial sensors, visual sensors, satellite navigation sensors, and so on [4]. As the heart and eyes of autonomous navigation systems, these sensors are intrinsic to the realization of autonomous and intelligent drones. However, satellite signals are interrupted by urban canyons and complex environments; in fog, heavy snow, and disaster scenarios, visual sensors lack sufficient feature points; inertial sensors face problems such as long-term error accumulation. Therefore, a single type of sensor alone cannot meet the autonomous navigation requirements of UAVs used in complex scenarios; multi-source sensors need to be used for fusion navigation. The multi-source fusion method can make up for the shortcomings of using a single type of sensor and establish complementary advantages and information supplementation between different sensors [5,6]. In this way, optimal estimations are obtained, and the reliability and real-time performance of an autonomous navigation system can be guaranteed.

Multi-source fusion localization algorithms include sequential filtering, decentralized filtering, centralized Kalman filtering, etc., [7,8]. As a distributed multi-source fusion filtering method, the federated Kalman method can facilitate plug-and-play in a multi-source fusion mode, thereby ensuring the navigational integrity and accuracy of the system. A. Carlson [9] proposed a two-stage distributed filtering federated Kalman filter algorithm, which includes N sub-filters, all of which are evenly distributed with information distribution coefficients.

However, in practical applications, the performance and estimation accuracy of a local system constantly change with the complex navigation environment, and the traditional Kalman filter information sharing coefficient is fixed, which means the different requirements of the navigation system cannot be met in complex scenarios. In order to improve the performance of the federated filter, Shen et al. [10] proposed a new adaptive federated Kalman filter with time-varying information sharing coefficients based on an observability analysis of the integrated navigation of unmanned ground vehicles. Xiong et al. [11] designed a novel dynamic vector-form information-sharing method based on an analysis of the error covariance matrix and the observation matrix of federated filters in highly dynamic environments. Zhang et al. [12] proposed a multi-source information fusion localization algorithm based on the federated Kalman filter, which has verified that the algorithm proposed in this paper displays fault tolerance and reduces the amount of required computation by comparing the centralized Kalman filter. Yue et al. [13] proposed an adaptive federated filtering algorithm that can calculate the information distribution coefficient using previous information and adjust the information distribution coefficient in real time. Lyu et al. [14] proposed an adaptive joint interactive multi-model (IMM) filter for complex underwater environments, which combines adaptive joint filtering with the IMM algorithm. Focusing on the problem of the variable accuracy of each navigation sensor, Guo et al. [15] designed an adaptive allocation algorithm of information factors based on prediction residuals. However, most of these studies focus on specific scenarios and the failure of a single sensor and lack discussions of different scenarios and different types of sensor failures. In this paper, we consider general adaptability. The proposed robust adaptive algorithm is not aimed at a single specific scene with a specific combination of sensors but is suitable for similar scenes with variable sensor combinations.

Focusing on the problems of GNSS signal interruption and the lack of sufficient feature points for visual sensors in complex scenes, this paper proposes a new robust adaptive positioning algorithm for UAV based on IMU/GNSS/VO, which can achieve the autonomous navigation and positioning of UAVs. Based on the ESKF, this paper establishes an integrated navigation model of IMU/GNSS and IMU/VO, incorporating system error model, measurement model and so on. Then, a robust adaptive localization algorithm is proposed based on a federated Kalman filter as the algorithmic framework, combined with robust equivalent weights and sub-filter adaptive shared coefficients. Finally, the time and accuracy of the three schemes are compared and analyzed through mathematical simulation experiments; the ‘OutBuilding’ scene data are selected, and the reliability and robustness of the proposed algorithm are verified through dataset tests.

This paper is organized as follows: A multi-source fusion model based on ESKF and federated Kalman filtering is established in Section 2, on the basis of the IMU/GNSS and IMU/VO integrated navigation model. In Section 3, an equivalent weight adaptive filtering algorithm is proposed based on robust equivalent weights and sub-filter adaptive shared coefficients. In Section 4, the accuracy and real-time performance of the three schemes are discussed and analyzed through mathematical simulation experiments. In Section 5, the effectiveness of the proposed algorithm is proven through dataset validation. Finally, the conclusions are drawn in Section 6.

## 2. Multi-Source Fusion Model

### 2.1. ESKF (Error State Kalman Filter)

Compared with the classical Kalman filter, the ESKF can constrain the error state to run at a position close to the origin, thereby avoiding the possible parameter singularity and gimbal lock problems and ensuring parameter linearization. In this paper, a loose combination of vision/inertial navigation and satellite/inertial navigation is modeled based on the ESKF. Similar to the classic Kalman approach, the ESKF performs prediction and measurement updates. The prediction model is kinematically updated based on the IMU (Inertial Measurement Unit) model, and the measurement is updated based on VO (Visual Odometry—the position and attitude data are obtained by solving camera image poses) and GNSS (Global Navigation Satellite Systems) measurement data.

#### 2.1.1. Predictive Model

This paper adopts the local navigation coordinate system, and the system state quantity is ${\left[q,v,p,a_{b},\omega_{b}\right]}^{Τ}$, where q represents UAV attitude quaternion, v represents UAV speed, p represents UAV position, $a_{b}$ represents the accelerometer bias, and $\omega_{b}$ represents the angular velocity bias. The UAV kinematics equation is as follows:

(1) $$\left\{\begin{array}{l} \dot{q}=\frac{1}{2}q⊗(\omega_{m}-\omega_{b})\\ \dot{v}=C_{b}^{n}(a_{m}-a_{b})\\ \dot{p}=\delta v\\ {\dot{a}}_{b}=0\\ {\dot{\omega }}_{b}=0 \end{array}\right.$$

Considering that the actual measurement contains errors, here, the state quantity is set to the error state $x(t)={\left[\delta \theta ,\delta v,\delta p,\delta a_{b},\delta \omega_{b}\right]}^{Τ}$. The UAV error state equation is as follows:

(2) $$\left\{\begin{array}{l} \delta \dot{\theta }={\left[\omega_{m}-\omega_{b}\right]}_{\times }\delta \theta -\delta a_{b}-w_{\omega }\\ \delta \dot{v}=-C_{b}^{n}{\left[a_{m}-a_{b}\right]}_{\times }\delta \theta -C_{b}^{n}\delta \omega_{b}-C_{b}^{n}w_{a}\\ \delta \dot{p}=\delta v\\ \delta {\dot{a}}_{b}=w_{a_{b}}\\ \delta {\dot{\omega }}_{b}=w_{\omega_{b}} \end{array}\right.$$

where δθ is the attitude angle error state that satisfies $\delta q=e^{\delta \theta /2}$, δv is the velocity error state, δp is the position error state, $\delta a_{b}$ is the accelerometer bias error state, and $\delta \omega_{b}$ is the angular velocity bias error state. $\omega_{m}$ is the measurement value of the gyroscope, $w_{\omega }$ is the measurement noise of the gyroscope, and $w_{\omega_{b}}$ is the noise of the gyroscope bias. $a_{m}$ is the measurement value of the accelerometer, $w_{a}$ is the measurement noise of the accelerometer, and $w_{a_{b}}$ is the noise of the accelerometer bias.

With reference to Equation (2), the equation for state is:

(3) $$\dot{x}(t)=F(t)x(t)+G(t)w(t)\;F(t)=\left[\begin{array}{ccccc} {\left[\omega_{m}-\omega_{b}\right]}_{\times } & 0_{3\times 3} & 0_{3\times 3} & -I_{3\times 3} & 0_{3\times 3}\\ -C_{b}^{n}{\left[a_{m}-a_{b}\right]}_{\times } & 0_{3\times 3} & 0_{3\times 3} & 0_{3\times 3} & -C_{b}^{n}\\ 0_{3\times 3} & 0_{3\times 3} & I_{3\times 3} & 0_{3\times 3} & 0_{3\times 3}\\ 0_{3\times 3} & 0_{3\times 3} & 0_{3\times 3} & 0_{3\times 3} & 0_{3\times 3}\\ 0_{3\times 3} & 0_{3\times 3} & 0_{3\times 3} & 0_{3\times 3} & 0_{3\times 3} \end{array}\right]\;G(t)=\left[\begin{array}{cccc} -I_{3\times 3} & 0_{3\times 3} & 0_{3\times 3} & 0_{3\times 3}\\ 0_{3\times 3} & -C_{b}^{n} & 0_{3\times 3} & 0_{3\times 3}\\ 0_{3\times 3} & 0_{3\times 3} & 0_{3\times 3} & 0_{3\times 3}\\ 0_{3\times 3} & 0_{3\times 3} & I_{3\times 3} & 0_{3\times 3}\\ 0_{3\times 3} & 0_{3\times 3} & 0_{3\times 3} & I_{3\times 3} \end{array}\right]w(t)=\left[\begin{array}{c} w_{a}\\ w_{\omega }\\ w_{a_{b}}\\ w_{\omega_{b}} \end{array}\right]$$

Using Taylor expansion, the formula is discretized, and the following formula is thus obtained:

(4) $$\begin{array}{l} x_{k+1}=(I+F\Delta T)x_{k}+G\Delta Tw_{k}\\ =\Phi_{k}x_{k}+\Gamma_{k}w_{k} \end{array}$$

where ΔT is the sampling time.

#### 2.1.2. Measurement Update

The UAV measurement update equation is as follows:

(5) $$z_{k}=H_{k}x_{k}+v_{k}$$

The GNSS measurement data are converted into the local navigation coordinate system of this paper, and the measurement matrix is obtained as follows:

(6) $$H_{k}^{GNSS}=[\begin{array}{ccccc} I_{3\times 3} & I_{3\times 3} & 0_{3\times 3} & 0_{3\times 3} & 0_{3\times 3} \end{array}]\;v_{k}={\left[\begin{array}{cc} n_{v}^{GNSS} & n_{p}^{GNSS} \end{array}\right]}^{Τ},n_{v}^{GNSS}\sim N(0,\sigma_{n_{v}^{GNSS}}^{2}),n_{p}^{GNSS}\sim N(0,\sigma_{n_{p}^{GNSS}}^{2})$$

where $n_{v}^{GNSS}$ is the velocity measurement white noise, and $n_{p}^{GNSS}$ is the white noise produced by position measurement.

Similarly, the VO measurement data are the position and attitude values obtained from the original image through pose calculation, and the measurement matrix is as follows:

(7) $$H_{k}^{VO}=[\begin{array}{ccccc} I_{3\times 3} & 0_{3\times 3} & I_{3\times 3} & 0_{3\times 3} & 0_{3\times 3} \end{array}]\;v_{k}={\left[\begin{array}{cc} n_{\theta }^{VO} & n_{p}^{VO} \end{array}\right]}^{Τ},n_{\theta }^{VO}\sim N(0,\sigma_{n_{\theta }^{VO}}^{2}),n_{p}^{VO}\sim N(0,\sigma_{n_{p}^{VO}}^{2})$$

where $n_{\theta }^{VO}$ is the attitude measurement white noise, and $n_{p}^{VO}$ is the position measurement white noise.

### 2.2. Fusion Model

**Figure 1:**
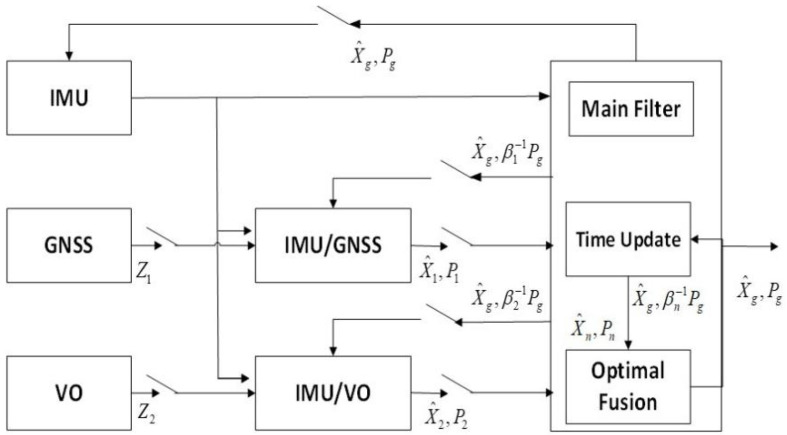
Federated Kalman filter.

The UAV measurement data are derived from two types of sensors, GNSS and VO, so the multi-source fusion method is used for state estimation. Considering the need to ensure the fault tolerance and reliability of the navigation system, the distributed filtering method is adopted in this paper. Figure 1 shows the classic fusion feedback mode of federated Kalman [16]. Two sub-filters are established using GNSS/IMU and VO/IMU, respectively, and finally the UAV navigation state is estimated by fusing the data of the two sub-filters.

#### 2.2.1. Time Update

The measurement update equation is as follows:

(8) $$\left\{\begin{array}{c} X_{k+1/k}^{i}=\Phi_{k+1/k}^{i}X_{k}^{i},i=1\cdots N,m\\ P_{k+1/k}^{i}=\Phi_{k+1/k}^{i}P_{k}^{i}\Phi_{k+1/k}^{i}{}^{Τ}+\Gamma_{k}^{i}Q_{k}^{i}{\left(\Gamma_{k}^{i}\right)}^{Τ} \end{array}\right.$$

where $X_{k}^{i}$ is the state quantity of the *i*-th (i=1⋯N) filter at time *k*, $X_{k}^{m}$ is the state quantity of the main filter at time *k*, $X_{k+1/k}^{i}$ is the one-step predicted state, $Q_{k}^{i}$ is the system state covariance, $\Phi_{k+1/k}^{i}$ is the state transition matrix of the *i*-th filter, and $P_{k+1/k}^{i}$ is the one-step predicted state covariance of the *i*-th filter.

#### 2.2.2. Measurement Update

The measurement update equation is as follows:

(9) $$\left\{\begin{array}{c} K_{k+1}^{i}=P_{k+1/k}^{i}{\left(H_{k+1}^{i}\right)}^{T}{\left(H_{k+1}^{i}P_{k+1/k}^{i}{\left(H_{k+1}^{i}\right)}^{T}+R_{k+1}^{i}\right)}^{-1}\\ X_{k+1}^{i}=X_{k+1/k}^{i}+K_{k+1}^{i}(Z_{k+1}^{i}-H_{k+1}^{i}X_{k+1/k}^{i})\\ P_{k+1}^{i}=(I-K_{k+1}^{i}H_{k+1}^{i})P_{k+1/k}^{i},i=1\cdots N \end{array}\right.$$

where $K_{k+1}^{i}$ is the gain matrix, $H_{k+1}^{i}$ is the measurement matrix, $R_{K+1}^{i}$ is the measurement state covariance, $X_{k+1}^{i}$ is the predicted state, and $P_{k+1}^{i}$ is the predicted state covariance.

#### 2.2.3. Information Fusion

The state quantity and state covariance of the main filter are obtained by fusing the sub-filters. The fusion equation is as follows:

(10) $$\left\{\begin{array}{c} P_{k+1}^{g}={\left[\sum_{i=1}^{N}{\left(P_{k}^{i}\right)}^{-1}\right]}^{-1},i=1\cdots N,m\\ X_{k+1}^{g}=P_{k+1}^{g}[\sum_{i=1}^{N}{\left(P_{k}^{i}\right)}^{-1}X_{k+1}^{i}] \end{array}\right.$$

where $P_{k+1}^{g}$ is the state covariance after the main filter fusion, and $X_{k+1}^{g}$ is the state quantity after the fusion of the main filter.

#### 2.2.4. Information Sharing and Feedback

The information sharing and feedback factor model is as follows:

(11) $$\left\{\begin{array}{c} Q_{k}^{i}=\beta_{i}{}^{-1}Q_{k}^{g}\\ \begin{array}{l} P_{k}^{i}=\beta_{i}{}^{-1}P_{k}^{g}\\ \sum_{i=1}^{N}\beta_{i}=1 \end{array}\\ X_{k}^{i}=X_{k}^{g},i=1\cdots N,m \end{array}\right.$$

where $\beta_{i}$ is the sub-filter sharing factor, and $\beta_{m}$ is the main filter sharing factor.

## 3. Robust Adaptive Filtering

In a complex environment, considering that errors, or even gross errors, arise in the measurement values of random dynamic systems, the statistical characteristics of noise will change, which will reduce the accuracy of Kalman filtering, and even cause divergence [15,17]. In this case, the availability of sub-filter data is reduced or even completely eliminated. One should consider performing residual testing and robustness processing on the sub-filters before the data fusion of the main filter in order to reduce the availability of observations. Unusable observations are isolated from the main filter so as not to contaminate the entire filtering process, thus improving the accuracy and fault tolerance of the entire system.

### 3.1. Robust Equivalent Weight Filtering

The system state residual is determined by both the model error and the observation error. When the model error is small, the residual can be used to represent the observation error, and the robustness equivalent weight factor can be used to alter the observation availability gain [18,19,20,21].

The state residual is $s_{k}^{i}=(z_{k}^{i}-H_{k}^{i}x_{k/k-1}^{i})$, and its covariance matrix is $w_{k}^{i}=H_{k}^{i}P_{k/k-1}^{i}H_{k}^{i}{}^{T}+R_{k}^{i}$.

Here, $s_{k}^{i}$ represents the residual of the *i*-th filter at time k in distributed filtering.

The residual of the *i*-th subfilter is normalized as follows:

(12) $$v_{i}={\left(s_{i}\right)}^{Τ}{{(w}_{i})}^{-1}s_{i}$$

Here, the IGG3 [22] weight function is introduced for robust processing, and the residual gain matrix is adaptively adjusted using the system’s normalized residual.

(13) $$\mu_{i}=\left\{\begin{array}{cc} 1 & \;|v_{i}|\leq k_{0}\;\\ (k_{0}/|v_{i}|\;)d_{{}_{i}}^{2} & {\;k}_{0}<|v_{i}|\;\leq k_{1}\\ 0 & \;|v_{i}|>k_{1} \end{array}\right.d_{i}=\frac{k_{1}-|v_{i}|}{k_{1}-k_{0}}$$

In the absence of gross errors in observations, the normalized residuals $v_{i}$ obey the standard state distribution: $v_{i}∼N(0,1)$. Robust processing is performed on observations that exceed the 95% confidence level, where $k_{0}$ is set to 1 and $k_{1}$ is set to 2. After the observation robustness is processed, the measurement update is performed as follows:

(14) $$\left\{\begin{array}{c} X_{k+1}^{i}=X_{k+1/k}^{i}+\mu_{i}K_{k+1}^{i}(Z_{k+1}^{i}-H_{k+1}^{i}X_{k+1/k}^{i})\\ {\bar{P}}_{k+1}^{i}=(I-\mu_{i}K_{k+1}^{i}H_{k+1}^{i})P_{k+1,k}^{i},i=1\cdots N \end{array}\right.$$

### 3.2. Adaptive Information Sharing Coefficient

In the classical federated Kalman, the sub-filters equally distribute the information sharing coefficient, i.e., $\beta_{1}=\ldots =\beta_{n}=1/n$ [17,23].

In practice, considering that the filtering accuracy of different sub-filters is inconsistent, it is necessary to adjust the proportion of information in each of the sub-filters according to the filtering accuracy. The information sharing coefficient determines the role of each sub-filter in the information fusion process. Specifically, the larger the information sharing coefficient, the larger the proportion of the state estimates dealt with by the local sub-filters [24].

In filtering, the state covariance $P_{i}$ positively reflects the filtering quality of the filter. The smaller the value of $P_{i}$, the more accurate the filter, and vice versa. Here, the accuracy of the sub-filter $\lambda_{i}(k)$ and the state covariance $P_{i}$ are defined by Equation (15), as follows.

(15) $$\lambda_{i}(k)=\sqrt{tr(P_{i}(k)\cdot P_{i}{\left(k\right)}^{Τ})}$$

As discussed in the previous section, the normalized residuals $v_{i}$ reflect the availability of filter observations, so we can combine $v_{i}$ and $P_{i}$ to comprehensively consider the accuracy of the sub-filters. Here, the IGG3 weight function is introduced to constrain the availability of observations. Considering Equations (13) and (15), the accuracy of the sub-filter can be determined as follows:

(16) $$\lambda_{i}(k)=\mu_{i}(k)\cdot \sqrt{tr({\bar{P}}_{i}(k)\cdot {\bar{P}}_{i}{\left(k\right)}^{Τ})}$$

given that in the federated Kalman filter, the information sharing coefficient satisfies [25,26,27,28]:

(17) $$\sum_{i=1}^{N}\beta_{i}(k)=1,0\leq \beta_{i}(k)\leq 1$$

where $\beta_{i}(k)$ is the information sharing coefficient of the *i*-th filter at step *k*.

Here, the main filter does not distribute information, so the adaptive information sharing coefficient and sub-filter precision $\lambda_{i}(k)$ are expressed as follows:

(18) $$\beta_{i}(k)=\frac{1/\lambda_{i}(k)}{1/\lambda_{1}(k)+1/\lambda_{2}(k)+\cdots +1/\lambda_{N}(k)},i=1,2,\cdots ,N$$

### 3.3. Robust Adaptive Multi-Source Model

**Figure 2:**
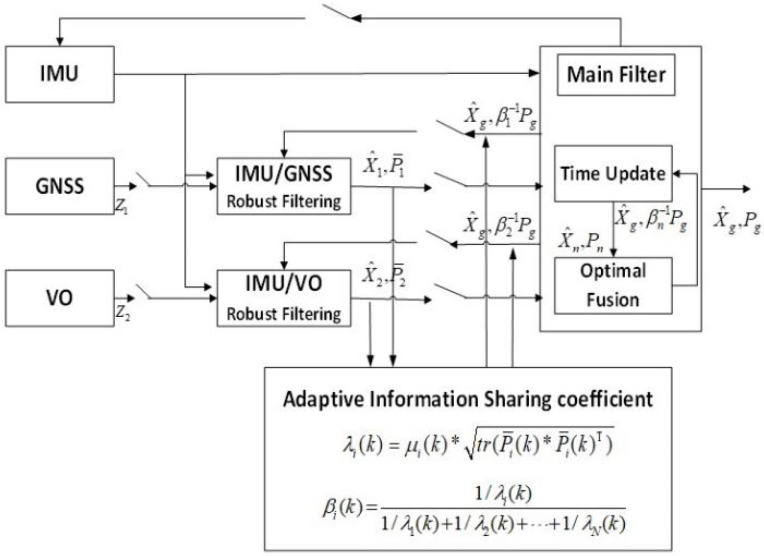
Robust adaptive multi-source model.

As shown in Figure 2, based on the federated Kalman filter, this study uses IMU/GNSS/VO to build a multi-source fusion navigation system. Robust filtering is performed on IMU/GNSS and IMU/VO, respectively, and the information sharing coefficients are adaptively adjusted by robust equivalent weights.

## 4. Simulation Experiment

In order to verify the effectiveness and robustness of the algorithm proposed in this paper, the parameters are set in alignment with the real characteristics of different sensors, and the scene is set with consideration for the complexity of the real environment. Simulation experiments are carried out to compare and analyze different schemes in different scenarios. We here highlight that the following simulation experiments have been developed and realized on the basis of the PSINS toolbox, completed by Prof. Yan Gongmin of Northwestern Polytechnical University.

### 4.1. Simulation Settings

#### 4.1.1. Track Settings

The simulation is set up with the initial position (local coordinates) as [0 m; 0 m; 0 m], the initial attitude (pitch, roll, yaw) as [0°; 0°; 0°], and the initial velocity (local coordinates) as [0 m/s; 0 m/s; 0 m/s]. The trajectory of the drone in the air is simulated, including acceleration, climbing, turning, descending, decelerating, and landing.

#### 4.1.2. Scene Settings

In consideration of the real urban environment, the challenging scenarios faced by UAV flight are here simulated. The following two periods are prone to measurement errors and have been designed considering the limitations of the motion model, and the complexity of the terrain and the environment.

Period 1: 100 s~200 s, when the UAV is flying between buildings; because there are few feature points, 20 times the $R_{VO}$ gross error is added to the VO positioning measurement. $R_{VO}$ is the measurement error value, including position error and attitude error, as shown in Table 1.

Period 2: 270 s~370 s, when the UAV flying height drops; here, the urban canyon environment is simulated, and 20 times $R_{GNSS}$ gross error is added to the GNSS positioning measurement. $R_{GNSS}$ is the measurement error value, including position error and speed error, as shown in Table 1.

#### 4.1.3. Sensor Parameter Settings

Table 1 shows the measurement error parameters and update frequency settings of each sensor (IMU, GNSS, VO).

#### 4.1.4. Simulation Scheme

In this paper, three schemes are designed to simulate the trajectory of the UAV in the above simulation.

Scheme 1: Traditional federated Kalman filtering. Information sharing coefficients are distributed equally.

Scheme 2: Adaptive federated Kalman filtering. The sub-filter remains unchanged, and the main filter adaptively adjusts the information sharing coefficient according to the accuracy of the sub-filter.

Scheme 3: Robust adaptive federated Kalman filtering. The sub-filter performs robust filtering, and the main filter adaptively adjusts the information sharing coefficient according to the accuracy of the sub-filter.

### 4.2. Experimental Results and Discussions

#### 4.2.1. Information Sharing Coefficient Simulation

The capacity of the information sharing coefficient for online adaptation can improve the accuracy and fault tolerance of the whole system in the case of partial sensor failure. The following shows a comparative analysis of the information sharing coefficients of the three schemes.

**Figure 3:**
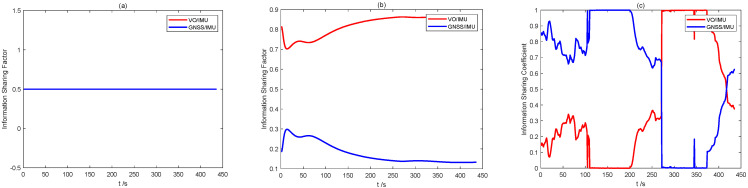
Comparison of information sharing coefficients of three schemes: (**a**) Scheme 1; (**b**) Scheme 2; and (**c**) Scheme 3.

Figure 3 shows the distribution of information sharing coefficients for the three schemes proposed in this paper. In Scheme 1, the sub-filter information sharing coefficients are evenly distributed. In Scheme 2, since the sub-filter observation error holds a fixed value, the mean square error shows a stable change trend, and the final factor weight of sub-filter information allocation is not changed. In Scheme 3, the information sharing coefficient shows a changing trend in periods 1 and 2. This is due to the presence of gross measurement or model errors, and the sub-filters worsen. The adaptive algorithm proposed in this paper can automatically reduce its corresponding information sharing coefficient and increase the sharing factors of the other two sub-filters. This is in line with expectations. Therefore, the algorithm can guarantee the fault tolerance of the whole system in a complex environment.

#### 4.2.2. Comparison of State Estimation of Different Combined Systems

**Figure 4:**
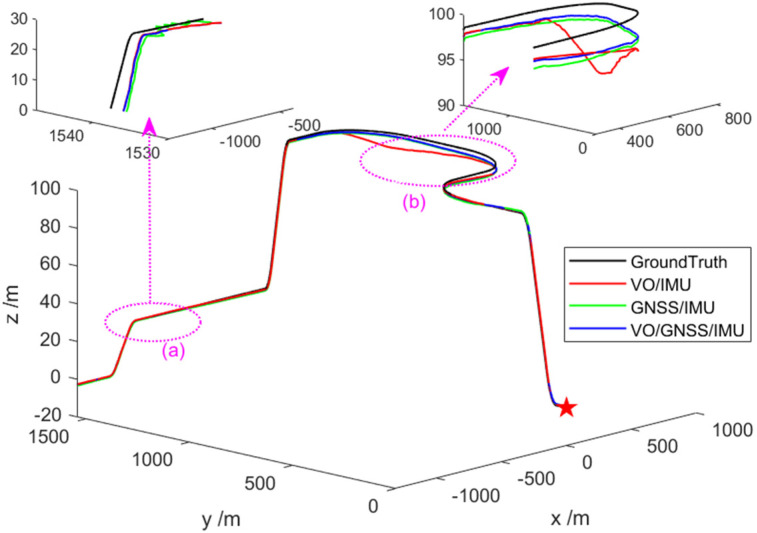
Comparison of state estimation of different combined systems. Asterisk indicates the starting position, the dotted line indicates the position of the enlarged area, and the arrow indicates the specific enlarged area (**a**,**b**).

In Figure 4, the black line represents the real trajectory, the red line represents the VO/IMU estimated trajectory, the green line represents the GNSS/IMU estimated trajectory, and the blue line represents the VO/GNSS/IMU estimated trajectory. If the three sub-filters are used for independent navigation, the state estimation accuracy will decrease due to the presence of gross errors in the observation values in different time periods (1, 2), which will cause a deviation from the true trajectory and mean the accuracy requirements of the entire navigation system are unmet. As expected, the performance of the VO/GNSS/IMU’s global optimal fusion is not seriously affected by abnormal signals given by local sensors and can achieve high accuracy.

#### 4.2.3. Comparison of the Results of Different Schemes

**Figure 5:**
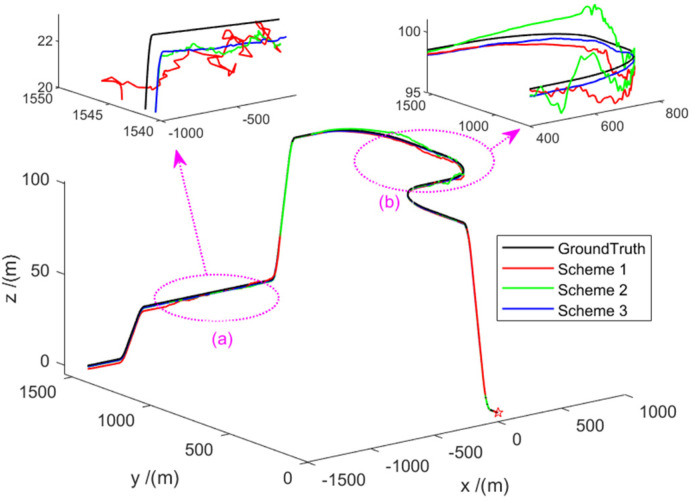
Comparison of trajectories of different schemes. Asterisk indicates the starting position, the dotted line indicates the position of the enlarged area, and the arrow indicates the specific enlarged area (**a**,**b**).

In Figure 5, the black line represents the true trajectory. The red line represents Scheme 1, which is the estimated trajectory of the federated Kalman filter. The green line represents Scheme 2, which is the estimated trajectory of the adaptive federal Kalman filter. The blue line represents Scheme 3, which is the estimated trajectory of the robust adaptive federal filtering proposed in this paper. It can be seen from Figure 5 that all three filtering methods can be used for VO/GNSS/GNSS system navigation. However, on the whole, and especially during the 100~200 s and 270~370 s periods, compared to the estimated trajectories of adaptive federated Kalman filtering and federated Kalman filtering, the estimated trajectories of the robust adaptive federal filter are the closest to the true values. This is because the robust adaptive federated Kalman can perform robust and adaptive adjustments on the sub-filter estimates, and the main filter performs adaptive information sharing coefficient allocation according to the estimated weights of the sub-filters. As expected, the performance of the main globally optimal fusion filter is not severely affected by local sensor anomalies and can achieve high accuracy.

**Figure 6:**
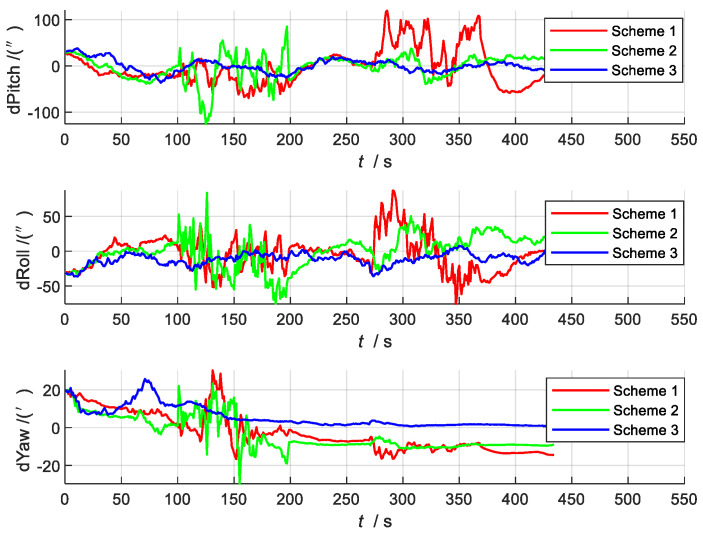
Comparison of attitude errors.

The attitude error estimation curve is shown in Figure 6. The attitude angle estimates obtained by the three filtering methods can all track the change in the true attitude angle, but their estimation accuracies are different. The error in the pitch angle and roll angle is within 100 arc seconds, and the error in the yaw angle is within 30 arc minutes. Compared with the actual data, the error here is small, which has a strong impact on the accuracy of the initial value and initial state covariance set by the simulation. At the same time, it can be seen that the accuracy of Scheme 3 is greatly improved compared with the other two schemes.

**Figure 7:**
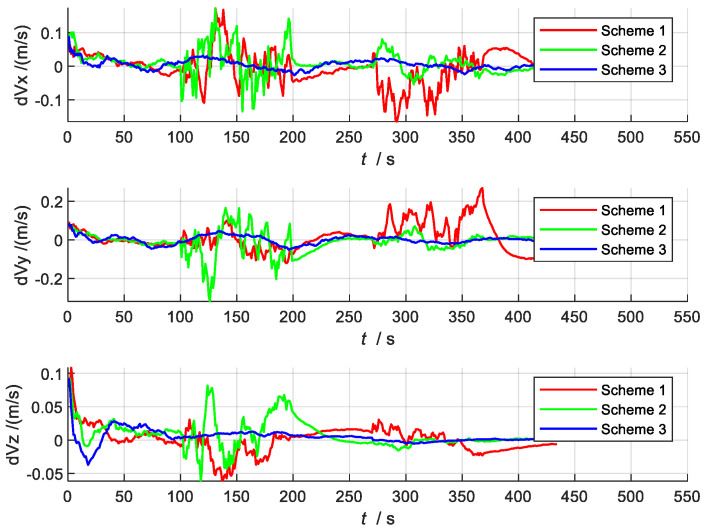
Speed error comparison chart.

**Figure 8:**
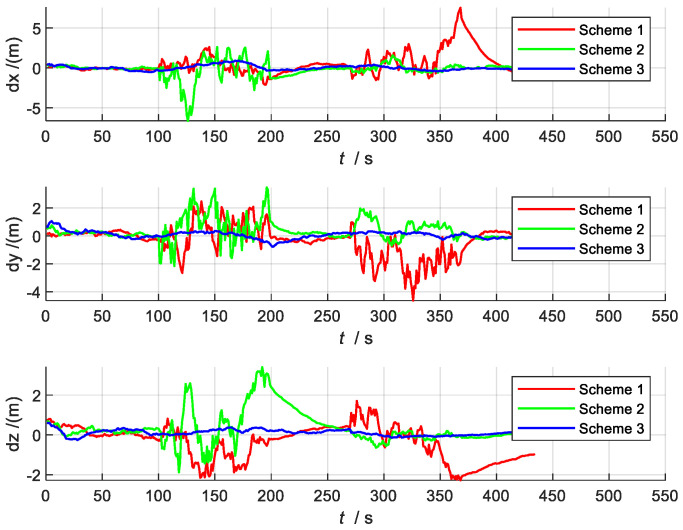
Comparison of position errors.

Figure 7 and Figure 8 show the comparison charts of speed and position error. According to the error estimation curve, it can be seen that the overall error of Scheme 3 is relatively stable, with a slight oscillation around the zero value. In the two time periods set in this paper, even when the local sensors are interfered with or fail entirely, the whole system can still maintain sufficiently high precision for navigation. This is because Scheme 3 can switch between different systems in time to reassign weights when local sensors are affected by external disturbances. Therefore, the robust adaptive filter can use the current adaptive state of each local system and can effectively utilize sub-filters with higher state accuracy, thereby reducing the estimated value of the error.

**Figure 9:**
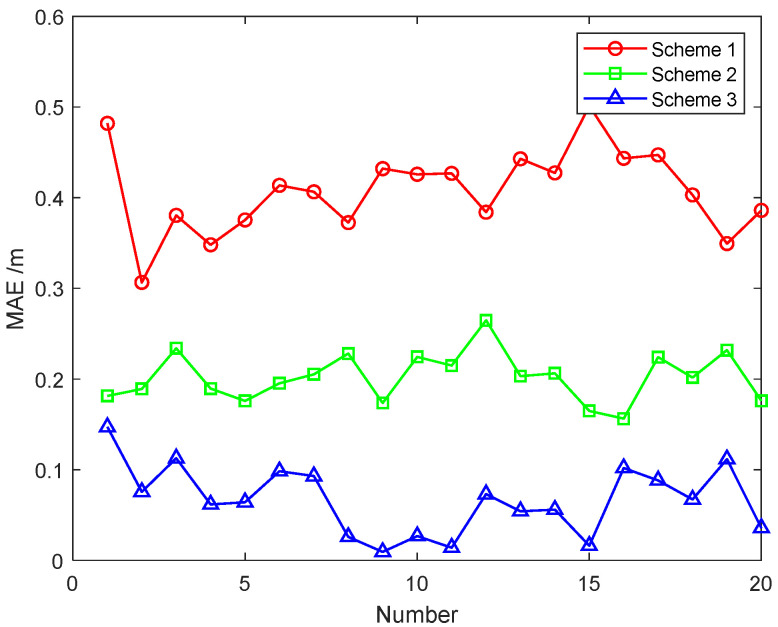
The MAEs of position errors (m) in the 20 experiment groups.

In order to further compare the three schemes used for information fusion, we carried out 20 Monte Carlo simulations analogous to the real environment. The noise, trajectory and speed of each setting are different. The mean absolute errors (MAEs) of the position errors for the 20 experiment groups are listed in Table 2, and the average error precision is shown in Figure 9.

As shown in Figure 9 and Table 2, the accuracy of Scheme 3 (robust adaptive Federated Kalman filtering) is significantly better than those of the other two schemes. The average position error accuracies of the 20 experiment groups were calculated separately, and the errors of the three schemes were obtained as follows: 0.4009 m, 0.2117 m, and 0.0719 m. Compared with Scheme 2, the accuracy of Scheme 3 increased by 66%, and compared with Scheme 1, it increased by 82%. The discussion and analysis of the above results further prove that the robust adaptive federated Kalman filtering algorithm proposed in this paper achieves high accuracy and good robustness, and the algorithm can be applied to complex environments.

The calculation times of the three algorithms have been tested. The test environment was Windows C++, and the test platform was configured at 1.99 GHz, with Intel(R) Core (TM) i7-8550U CPU. The times required for the single-step execution of the three schemes are shown in Table 3. The time required for the single-step execution of the robust adaptive federated Kalman filter algorithm was 2.12 × 10^−2^, which meets the real-time requirements of practical applications.

## 5. Dataset Validation

**Figure 10:**
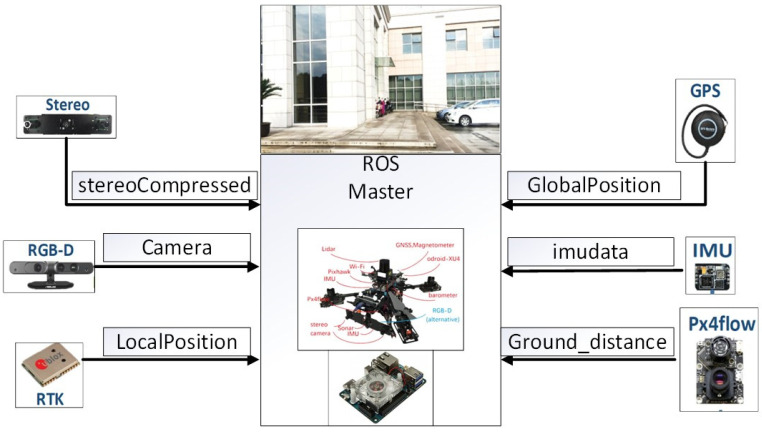
UAV sensor configuration (different sensors and mounting locations for UAV) and “OutBuilding”.

On the basis of the simulation verification preformed in the previous section, the dataset collected by the Shanghai Beidou Navigation and Location Services Key Laboratory (UAV configuration sensors and related parameters are shown in Figure 10 and Table 4) are used for verification. This dataset includes four scenarios: 5 × 5 × 2.5 m testing room with Vicon, “Room”; 8 × 12 × 5 m hall of office with Vicon, “Hall”; 20 × 20 m outdoor square, “OutSquare”; 50 m^2^ outdoor area near the building, “OutBuilding”. Among the four scenarios, “OutBuilding” (“OutBuilding” is shown in Figure 10) is the most representative, and offers the conditions of short-term errors in or interruptions of GNSS and VO measurement due to signal occlusion or single features. In order to test the applicability of the algorithm in this paper in a complex environment, the SE_OutBuilding_06.bag data are here used to artificially add errors in different time periods. By comparing the final results of the three different schemes, the effectiveness of the algorithm in this paper is verified.

**Figure 11:**
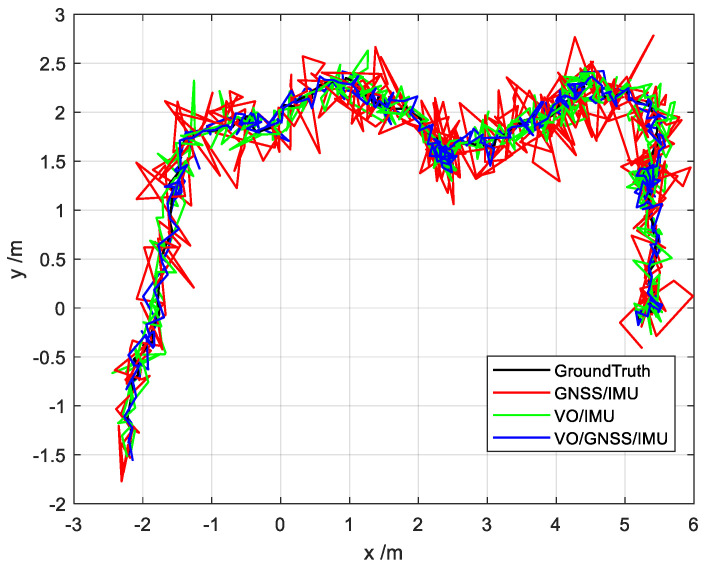
Location estimation for different scenarios.

First, based on the ESKF model, the state estimation results of the VO/IMU, GNSS/IMU, and VO/GNSS/IMU integrated navigation systems are obtained, as shown in Figure 11. It can be seen that these integrated navigation systems meet the needs of navigation and positioning, without model or measurement errors. The positioning accuracy is determined by the accuracy and combination of the sensors themselves. The VO/GNSS/IMU combination shows the highest accuracy, followed by the VO/IMU combination, and finally GNSS/IMU.

In consideration of the real properties of the sensor and the complex external environment, the following two time periods are set. These two periods contain model and measurement errors, which can enable us to more effectively verify the algorithm proposed in this paper.

Period 1: 10~40 s; non-Gaussian noise is added to RGB-D Camera measurement, which obeys the following distribution:

$$g(\omega )=\frac{1-\varepsilon }{\sigma_{1}\sqrt{2\pi }}\exp(-\frac{\omega^{2}}{2\sigma_{1}^{2}})+\frac{\varepsilon }{\sigma_{2}\sqrt{2\pi }}\exp(-\frac{\omega^{2}}{2\sigma_{2}^{2}})$$

where $\sigma_{1}=2500\;\mu \mathrm{rad}$, $\sigma_{2}=4\sigma_{1}$ and ε=0.5.

Period 2: 60~90 s; 20R random error is added to RTK GNSS receiver positioning measurement.

**Figure 12:**
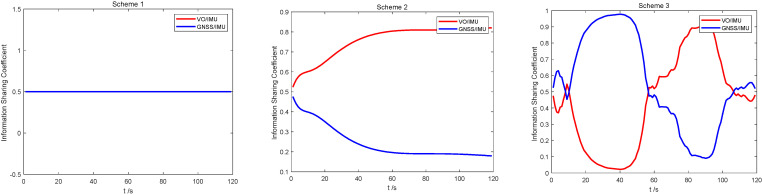
Information sharing coefficient of different schemes (Scheme 1, Scheme 2, Scheme 3).

As shown in Figure 12, the information sharing coefficients of different schemes show different trends, as consistent with the simulation results in Section 4.2. Since the measurement accuracy of the vision sensor is higher than that of GNSS, when the information sharing coefficient in Scheme 2 stabilizes, the ratio of VO/IMU will be higher. At the same time, the information sharing coefficient of Scheme 3 shows a change trend, which indicates that the information sharing coefficient of the robust adaptive equivalent Kalman filter algorithm can be adjusted online when the environment changes, thereby improving the accuracy of the entire system.

**Figure 13:**
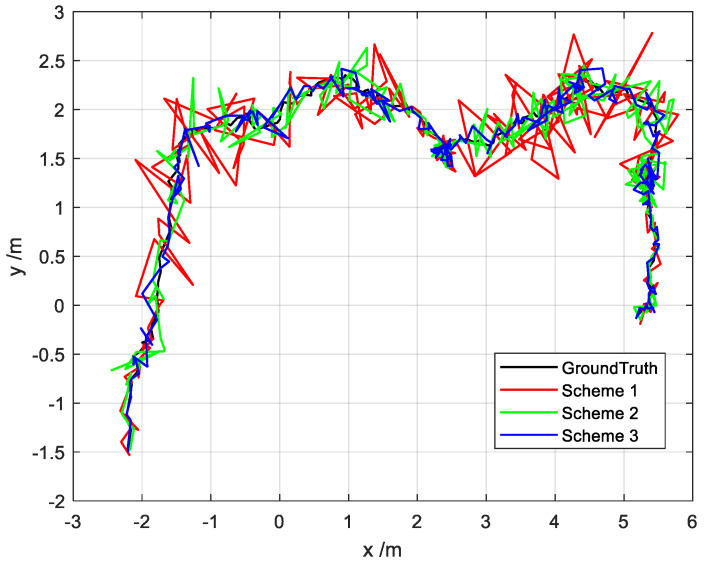
Position estimation for different scenarios.

Using the three schemes set in Section 4.1, a position estimate is obtained as shown in Figure 13. Here, the black line represents the true trajectory; the red line (Scheme 1) represents the estimated trajectory of the federated Kalman filter; the green line (Scheme 2) represents the estimated trajectory of the adaptive information sharing coefficient of the main filter; the blue line (scenario 3) represents the estimated trajectory of the robust adaptive federated filter proposed in this paper. As can be seen, the robust adaptive federated Kalman filter proposed in this paper can effectively track the ground truth.

**Figure 14:**
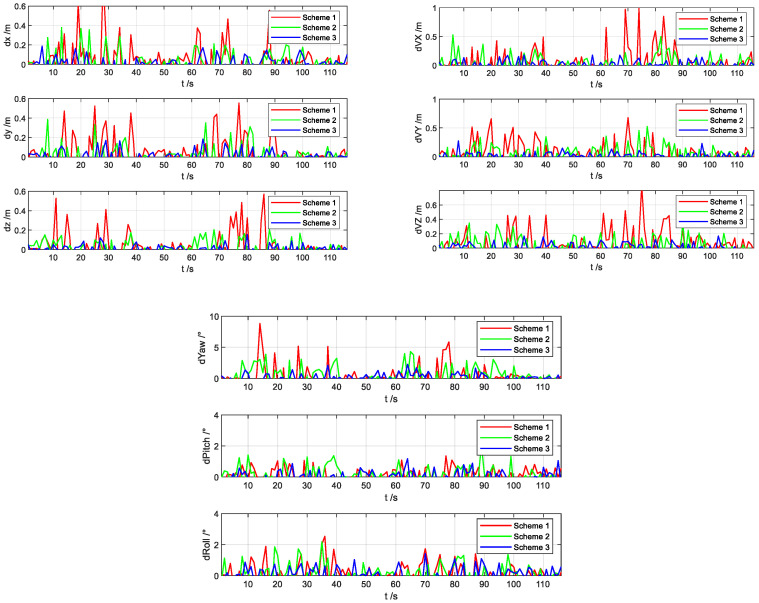
Comparison of position, speed, and attitude errors of different schemes.

Figure 14 shows a comparison of the position, velocity, and attitude errors of the three schemes. Compared with Scheme 1 and Scheme 2, Scheme 3 has a higher overall accuracy, which is consistent with the simulation results shown in Section 4.2. For the next 20 analyses, the mean absolute errors (MAEs) and standard deviations (STDs) of the state estimation errors of the three schemes are obtained individually, as shown in Table 5.

As can be seen from Table 5, compared with Scheme 1 and Scheme 2, the average value of the pitch angle and roll angle in Scheme 3 is increased by 1 degree, and the average value of the yaw angle is increased by 2 degrees. The average speed is increased by 0.2 m/s, and the average position is increased by about 0.2 m. These experimental results further demonstrate that the robust adaptive Kalman filter algorithm proposed in this paper can effectively improve the accuracy and robustness of the multi-source fusion navigation system. Scheme 3 is significantly better than the other two schemes, with an overall accuracy improvement of 2–3-fold.

## 6. Conclusions

With the intention of improving the reliability and robustness of UAV autonomous navigation and positioning in complex scenarios, we have here designed an autonomous positioning fusion algorithm. The main innovation is that the algorithm can not only independently evaluate the working performance of the sub-filters online, but it can also dynamically adjust the information sharing coefficient. In order to verify the effectiveness and robustness of the algorithm proposed in this paper, an urban canyon scene has been simulated. Through comparative analysis of the two scenarios and three schemes set up, Scheme 3 displayed the highest accuracy of robust adaptive federal kalman filtering, followed by Scheme 2 (adaptive federal Kalman filtering), and finally Scheme 1 (federal Kalman filtering). In addition, by testing the time taken for the single-step debugging of the robust adaptive federal Kalman filter, it has been proven that the algorithm can meet the requirements of actual real-time measurements. Further, this paper used the Beidou Navigation and Location Services Key Laboratory dataset for verification. Using the “OutBuilding” data, the artificially simulated model errors and measurement gross errors have been added, and the final results show that the overall accuracy of the algorithm proposed in this paper is improved 2–3-fold. In summary, the algorithm can significantly improve the accuracy and tolerance of the navigation system in complex environments and can be applied to UAV autonomous navigation in urban canyons and GNSS loss-of-lock scenarios. Moreover, the algorithm is general, and can be applied in similar complex scenes and other sensor combinations. Therefore, using the robust adaptive fusion algorithm proposed in this paper, reliable, adaptive, robust and high-precision positioning information can be obtained. Next, we will focus on the actual application of UAVs in complex environments to verify the effectiveness of the algorithm proposed in this paper.

## Figures and Tables

**Table 1:** Sensor parameter settings.

Sensor Type	Parameter	Value
IMU	Gyro bias error	$0.1{}^{{}^{\circ}}/h$
	Gyro random walk error	$0.08{}^{{}^{\circ}}/\sqrt{h}$
	Accelerometer bias error	200 μg
	Accelerometer random walk error	$50\;\mu g/\sqrt{h}$
	Frequency	100 Hz
GNSS	Position error	[1 m; 1 m; 3 m]
	Speed error	[0.1 m/s; 0.1 m/s; 0.1 m/s]
	Frequency	1 Hz
VO	Position error	[0.5 m; 0.5 m; 0.5 m]
	Attitude error	[0.5°; 0.5°; 0.5°]
	Frequency	2 Hz

**Table 2:** The MAEs of position errors (m) in the 20 experiment group.

Number	Scheme 1	Scheme 2	Scheme 3
1	0.4818	0.1813	0.1470
2	0.3065	0.1891	0.0755
3	0.3805	0.2339	0.1125
4	0.3480	0.1894	0.0616
5	0.3754	0.1758	0.0641
6	0.4136	0.1953	0.0983
7	0.4064	0.2052	0.0929
8	0.3724	0.2280	0.0258
9	0.4319	0.1735	0.0093
10	0.4257	0.2243	0.0267
11	0.4267	0.2148	0.0140
12	0.3838	0.2646	0.0730
13	0.4428	0.2031	0.0542
14	0.4273	0.2063	0.0559
15	0.5012	0.1648	0.0162
16	0.4434	0.1562	0.1018
17	0.4471	0.2241	0.0882
18	0.4030	0.2017	0.0671
19	0.3493	0.2318	0.1115
20	0.3860	0.1759	0.0357

**Table 3:** The time required for the single-step execution of the three schemes.

	Scheme 1	Scheme 2	Scheme 3
Time (s)	7.56 × 10^−3^	9.01 × 10^−3^	2.12 × 10^−2^

**Table 4:** Sensors and related parameters.

Sensor	Product Model	Collection Frequency (Hz)
Optical flow	Px4flow v1.3.1	20
Stereo camera	640 × 480 × 2 OV7725	30
IMU	MPU9250	40
RGB-D Camera	ASUS Xtion Pro Live	40
Vicon	Vero 360	100
RTK GNSS receiver	Ublox M8P	10

**Table 5:** Accuracy statistics of different schemes (the mean absolute errors (MAEs) and standard deviations (STDs) of the state estimation errors of the three schemes).

Error		Pitch (°)	Roll (°)	Yaw (°)	VX (m/s)	VY (m/s)	VZ (m/s)	X (m)	Y (m)	Z (m)
Scheme 1	MAE	1.56	1.62	3.01	0.30	0.25	0.22	0.26	0.24	0.19
	STD	0.95	0.97	1.97	0.23	0.22	0.19	0.50	0.55	0.64
Scheme 2	MAE	1.08	1.12	1.78	0.15	0.14	0.12	0.13	0.12	0.11
	STD	0.81	0.85	1.25	0.15	0.11	0.09	0.32	0.45	0.84
Scheme 3	MAE	0.62	0.55	0.95	0.07	0.07	0.06	0.06	0.07	0.06
	STD	0.41	0.23	0.52	0.10	0.08	0.07	0.15	0.18	0.14

## Data Availability

Not applicable.
